# Physiological associations with heart rate–speed decoupling during a half-marathon in adolescent endurance runners

**DOI:** 10.3389/fphys.2026.1807399

**Published:** 2026-04-29

**Authors:** Weiyu Wang, Ruihang Zhou, Guangze He, Yumeng Si, Yongqin Cao, Pengxi Zheng, Wei Zi

**Affiliations:** Sports Training Research Center, China Institute of Sport Science, Beijing, China

**Keywords:** adolescent endurance runners, durability, energy supply regulation, heart rate–speed decoupling, thermoregulation

## Abstract

**Introduction:**

From a process-oriented perspective of endurance durability, this exploratory study aimed to characterize the dynamic evolution of heart rate–running speed decoupling during a simulated half-marathon in adolescent endurance athletes and to examine phase-specific associations between decoupling characteristics and physiological regulation.

**Methods:**

Thirteen adolescent endurance runners with systematic endurance training completed a 21.1-km simulated half-marathon under self-paced conditions. Heart rate and running speed were recorded continuously and aggregated into consecutive 2-km distance segments. Internal and external loads were normalized to individual maximal heart rate and running speed at VO_2peak_, respectively, and used to derive a relative heart rate–running speed decoupling index (DM_i_). Decoupling characteristics included the distance of decoupling onset (Onset), maximal decoupling magnitude (DM_max_), and mean decoupling during the late phase of exercise (15–20 km). Core body temperature, interstitial glucose concentration, and sweat-derived estimates of fluid and sodium loss were monitored concurrently. Associations were examined using Spearman correlation analysis.

**Results:**

Heart rate–running speed decoupling increased progressively throughout the run and showed substantial inter-individual variability. The median decoupling onset occurred at 10.0 km (interquartile range: 7.0–12.0 km). An earlier onset was associated with greater concurrent increases in core body temperature (ρ = −0.579, *p* = 0.038) and sodium loss per unit distance (ρ = −0.605, *p* = 0.037). The maximal decoupling magnitude (DM_max_) was positively correlated with changes in core body temperature (ρ = 0.632, *p* = 0.021) and sodium loss per unit distance (ρ = 0.643, *p* = 0.024). In contrast, the change in decoupling during the final stage of the run (15–20 km) was not associated with thermoregulatory or fluid–electrolyte indices but was positively correlated with changes in glucose concentration (ρ = 0.599, *p* = 0.031).

**Discussion:**

During a simulated half-marathon, heart rate–running speed decoupling evolved progressively in adolescent endurance athletes and demonstrated marked inter-individual variability. Decoupling onset and maximal magnitude were primarily associated with thermoregulatory and electrolyte-related physiological loads during the early to middle stages of exercise, whereas decoupling dynamics in the latter stage were more closely aligned with physiological responses related to energy supply regulation. These findings support heart rate–running speed decoupling as a durability-related indicator with phase-specific physiological characteristics during prolonged endurance exercise.

## 1 Introduction

The half-marathon is a typical prolonged endurance event, during which athletes are not exposed to a constant workload throughout the race. Instead, runners continuously adjust their pacing strategies in response to perceptual feedback, tactical considerations, and environmental conditions, resulting in pronounced dynamic fluctuations in external load (e.g., running speed), while internal physiological load progressively accumulates with increasing exercise duration and distance (Cheuvront and Haymes, 2001). Under such conditions, describing endurance race load solely on the basis of mean exercise intensity or isolated time-point measures is insufficient to fully capture the dynamic coupling between internal and external loads across the entire competition. At present, the relationship between heart rate and running speed is widely used as an indicator of internal–external load coupling during endurance exercise. When physiological regulation is relatively stable, heart rate generally maintains a consistent correspondence with running speed. However, during prolonged endurance exercise, heart rate may progressively increase over time even when running speed remains relatively stable, manifesting as heart rate–running speed decoupling or cardiovascular drift (Coyle and González-Alonso, 2001; Coyle, 1998; Montain and Coyle, 1992). Previous studies have shown that this phenomenon frequently coincides with dehydration, elevations in core body temperature, and increased circulatory regulatory strain, and has therefore been proposed as a marker describing the time-dependent divergence of internal load relative to external load (Coyle and González-Alonso, 2001; Montain and Coyle, 1992).

In recent years, with the emergence of the concept of endurance performance “durability,” internal–external load decoupling has increasingly been used to characterize athletes’ physiological capacity to maintain performance output under prolonged loading conditions and has been applied to process-based analyses of endurance events such as the marathon (Impellizzeri et al., 2019; Smyth et al., 2022). Importantly, heart rate–running speed decoupling is unlikely to reflect the action of a single physiological system, but rather represents the integrated manifestation of multiple regulatory processes. During prolonged endurance exercise, thermal strain leads to progressive elevations in core body temperature, thereby increasing the cardiovascular regulatory burden associated with heat dissipation (González-Alonso et al., 1999; Nybo, 2008; González-Alonso, 2012; Périard et al., 2021). Metabolic strain is reflected by dynamic changes in blood glucose concentration, representing the balance between energy supply and utilization, and dysregulation of glucose homeostasis may further impair performance and contribute to central fatigue (Coyle, 1995; Jeukendrup, 2004). Concurrently, fluid and electrolyte losses through sweating and sodium excretion may reduce plasma volume and alter neurohumoral regulation, thereby exacerbating cardiovascular strain (Shirreffs and Sawka, 2011; Baker, 2017; Baker et al., 2022; Anastasiou et al., 2009). These physiological processes occur simultaneously during endurance competition and may interact to shape the physiological responses observed in the latter stages of prolonged endurance events. However, existing studies have largely focused on single physiological markers or have been conducted under constant-load laboratory conditions, with limited attention given to the process-oriented dynamics of multiple physiological variables (e.g., core temperature, glucose, and fluid–electrolyte balance) in real or near-real race scenarios. Moreover, current evidence regarding heart rate–running speed decoupling and its underlying regulatory mechanisms is derived primarily from adult endurance athletes or healthy adult populations. In contrast, the physiological regulatory systems of adolescent endurance athletes are still undergoing development and maturation, and their cardiovascular regulation, thermoregulatory capacity, metabolic responses, and fluid balance mechanisms may differ substantially from those of adults (Armstrong and Barker, 2011; Falk and Dotan, 2008). Previous research has demonstrated that adolescents exhibit distinct physiological characteristics compared with adults, particularly in terms of sweating capacity, heat dissipation efficiency, and energy metabolism strategies (Smallcombe et al., 2025). Therefore, directly extrapolating findings from adult endurance research to adolescent populations may underestimate or misinterpret adolescents’ physiological responses to prolonged endurance loading. At present, systematic investigations examining internal–external load decoupling alongside multiple physiological responses throughout an entire endurance race in adolescent athletes remain scarce.

Based on this background, the present study investigated adolescent endurance runners under a simulated half-marathon race condition using a process-oriented research design, with continuous monitoring of heart rate, running speed, core body temperature, glucose concentration, and fluid–electrolyte-related indices throughout the run. The aim of this study was to (1) characterize the dynamic features of internal–external load decoupling during a simulated half-marathon in adolescent athletes, and (2) examine the associations between decoupling characteristics and multiple physiological variables, including core temperature, glucose, and fluid–electrolyte regulation. As an exploratory analysis, this study sought to provide novel insights into the physiological regulatory characteristics of adolescent endurance athletes under prolonged endurance loading conditions.

## 2 Methods

### 2.1 Participants

This study recruited adolescent endurance athletes as participants. A total of 13 athletes were included, all of whom had a history of systematic endurance training (defined as >2 years of regular training) and were capable of completing a half-marathon distance. During the study period, all participants were in good health, with no apparent injuries or medical conditions that could affect endurance performance.

Basic characteristics of the participants, including age, height, body mass, and maximal oxygen uptake (VO_2max_), are presented in **Table 1**. The study protocol was reviewed and approved by the Science and Technology Ethics Committee (Approval No. 20260129), and written informed consent was obtained from all participants and their legal guardians prior to participation.

**Table 1:** Participant characteristics (*n* = 13).

Variable	Median (IQR)
Age (years)	16 (15–17)
Height (cm)	172 (164–178)
Body mass (kg)	55 (48–58)
VO_2max_ (mL·kg^-1^ min^-1^)	68.4 (60.8–71.3)
Training experience (years)	3 (2–3)

### 2.2 Study design

This study was designed to examine performance characteristics under a simulated race condition. Participants completed two testing sessions on separate days, including a laboratory-based physiological baseline test and a simulated half-marathon run, with a minimum interval of 48 h between sessions to minimize the potential influence of maximal exercise testing on subsequent simulated race performance and physiological responses. During the simulated race, external load variables (running speed and distance) and internal load variables (heart rate) were recorded concurrently, while core body temperature, glucose concentration, and fluid–electrolyte-related indices were monitored continuously throughout the run. Environmental conditions (including ambient temperature, relative humidity, and wind speed) were recorded at 15-min intervals on each test day and are presented as descriptive contextual information in **Supplementary Table 1**. These variables were not included in the primary statistical analyses because the study was designed as a small-sample, process-oriented exploratory analysis, and the variability in environmental conditions across test days was relatively limited.

### 2.3 Physiological baseline testing

To obtain baseline physiological characteristics related to endurance performance, all participants completed an incremental treadmill running test under laboratory conditions. The test was performed on a motorized treadmill (h/p/cosmos), with oxygen uptake (VO_2_) and carbon dioxide production (VCO_2_) measured continuously using a cardiopulmonary exercise testing system (MAX-II). Heart rate was recorded concurrently via a chest-strap monitor (Polar H10, Finland) (Merrigan et al., 2022). Prior to each test, the gas analyzers and flow sensors were calibrated according to the manufacturer’s specifications. Participants were instructed to refrain from high-intensity exercise for at least 48 h before testing. Each test session began with a standardized warm-up (~15 min), followed by 3 min of quiet standing on the treadmill. The initial treadmill gradient was set at 1% (Jones and Doust, 1996). The test employed a stepwise incremental protocol. Initial running speed was set at 7 km·h^-1^ for women and 9 km·h^-1^ for men, with speed increased by 1.6 km·h^-1^ every 2 min until a speed of 24 km·h^-1^ was reached (Machado et al., 2013). If volitional exhaustion had not occurred at this speed, the workload was further increased by progressively raising the treadmill gradient by 1° per minute while maintaining running speed, until volitional exhaustion.

Peak oxygen uptake (VO_2peak_) was defined as the highest 15-s averaged VO_2_ value attained during the test. VO_2peak_ was considered VO_2max_ when at least two of the following criteria were met: respiratory exchange ratio (RER) ≥ 1.10 and rating of perceived exertion (RPE) ≥ 19 (Howley et al., 1995). The running speed corresponding to VO_2peak_ (vVO_2peak_) was determined by performing linear regression analysis between steady-state VO_2_ values (averaged over the final 30 s of each stage between 8 and 24 km·h^-1^) and running speed (VO_2_ = *a*·*v* + *b*). The VO_2peak_ value was subsequently substituted into the regression equation to calculate vVO_2peak_.

### 2.4 Simulated half-marathon run protocol

The simulated half-marathon run was conducted on a calibrated 5-km looped course. Participants completed four full laps plus a final 1.1-km segment to cover the total distance of 21.1 km. The course was flat and performed under the direct supervision of the research staff. Participants were instructed to adopt a self-paced running strategy based on their individual race plans, in order to closely replicate real-world race conditions, consistent with pacing patterns observed in endurance competitions (Nikolaidis et al., 2019).

No fixed aid stations were provided during the run, and no additional fluid intake was permitted. Environmental conditions, including ambient temperature and wind speed, were recorded on the test day for contextual reference.

### 2.5 Physiological measurements and data processing

#### 2.5.1 Heart rate–running speed decoupling indices

Heart rate and running speed data were collected continuously throughout the simulated half-marathon run using a GPS-enabled sports watch (Garmin Forerunner 965) and a chest-strap heart rate monitor (Garmin HRM Pro Plus), which has shown strong agreement with electrocardiogram (ECG)-derived heart rate measures in previous validation studies of similar Garmin devices (Merrigan et al., 2022). Data were exported on a per-kilometer basis, subsequently organized by distance, and further aggregated into 2-km distance segments for statistical analysis. Based on maximal heart rate (HR_max_) and the running speed corresponding to maximal oxygen uptake (vVO_2peak_) obtained from the incremental treadmill test, heart rate and running speed values for each distance segment were normalized on an individual basis to derive indices of relative internal and external load.

Relative internal load for each segment (In_seg_) was defined as the mean heart rate expressed as a percentage of maximal heart rate:


$${\text{In}}_{\text{seg}}=\frac{{\text{HR}}_{\text{seg}}}{{\text{HR}}_{\text{max}}}\;\times \;100\%$$

Relative external load for each segment (Ex_seg_) was defined as the mean running speed expressed as a percentage of vVO_2peak_:


$${\text{Ex}}_{\text{seg }}=\frac{{\text{V}}_{\text{seg}}}{{\text{V}}_{{\text{VO}}_{2\text{peak}}}}\;\times \;100\%$$

On this basis, the relative internal–external load ratio for each distance segment (R_seg_) was calculated as:


$${\text{R}}_{\text{seg}}\;=\frac{{\text{In}}_{\text{seg}}}{{\text{Ex}}_{\text{seg}}}$$

To characterize changes in internal load relative to external load across the race, the 3- to 4-km distance segment (2-km length) was selected as the reference segment. This segment was selected as an operational early-race reference phase to minimize pacing-related fluctuations typically observed at race onset and to represent a stage in which running rhythm was expected to be more stable than during the start phase, consistent with previous observations on pacing behavior in endurance running (Casado et al., 2021; Ristanović et al., 2023). This choice was further supported by the present dataset, in which within-segment speed variation was generally smaller in the 3- to 4-km segment than in the initial 1- to 2-km segment (**Supplementary Table 2**). Using this reference segment, the relative internal–external load ratio for each distance segment was normalized to derive the heart rate–running speed decoupling index (DM_i_):


$${\text{DM}}_{\text{i}}=\frac{{\text{R}}_{\text{seg}}}{{\text{R}}_{\text{ref}}}$$

where R_ref_ represents the relative internal–external load ratio during the reference segment (3–4 km). A DM_i_ value greater than 1 indicates an increased heart rate response at a given relative external intensity compared with the early-race reference condition. It should be noted that DM_i_ is a ratio-derived, within-individual normalized index and may therefore be influenced by measurement variability in both the numerator and denominator, as well as by the stability of the reference segment used for normalization. Accordingly, DM_i_ should be interpreted as a relative indicator of internal–external load divergence within an individual rather than as an absolute physiological quantity (Allison et al., 1995; Curran-Everett, 2013; Holmes and Buhr, 2007). Based on the DM_i_ profile across the race, the following decoupling characteristics were extracted for process-based analyses:

DM_max_ (maximal decoupling magnitude): the maximum DM_i_ value observed across the entire race, reflecting the greatest degree of internal–external load divergence reached during the run.

Onset (decoupling onset) was operationally defined as the first race distance at which DM_i_ exceeded 1.025 for at least two consecutive 2-km segments. The 2.5% threshold was selected as a conservative criterion to identify sustained divergence in relative internal versus external load, rather than transient variation around the reference segment, in line with previous work on durability-related decoupling (Smyth et al., 2022; Hunter et al., 2025).

DM_mean-late_ (mean late-race decoupling): the mean DM_i_ value calculated over the final phase of the race (15–20 km), used to reflect the overall burden of decoupling during the latter stage of the simulated half-marathon.

#### 2.5.2 Core temperature, glucose, and fluid–electrolyte measures

During the simulated half-marathon run, core body temperature, glucose concentration, and fluid–electrolyte-related indices were monitored continuously. All physiological data were time-stamped and synchronized with running performance data, and subsequently organized according to race distance. This approach enabled process-oriented analyses based on the temporal sequence of decoupling onset and race-stage-specific responses. To examine physiological changes around decoupling onset, phase windows were defined relative to each athlete’s individual Onset. The pre-decoupling phase was defined as the 3 km preceding Onset, whereas the decoupling phase was defined as Onset ± 1 km. A 1-km overlap was intentionally retained to preserve temporal proximity to the onset event and to ensure sufficient data density for continuously monitored variables. Accordingly, the resulting Δ_at–pre_ indices should be interpreted as process-oriented transition measures rather than strictly independent pre–post contrasts.

##### 2.5.2.1 Core body temperature

Core body temperature was continuously monitored throughout the simulated half-marathon run using a wearable core temperature monitoring device (CORE Body Temperature Sensor, USA), which provides dynamic estimates of core temperature based on skin temperature and heat flux signals. Previous studies have validated this device against reference measures such as rectal temperature under both exercise and heat-stress conditions, demonstrating that it is capable of reasonably capturing temporal changes in core body temperature, although systematic bias may occur depending on environmental conditions and individual characteristics (Vedel et al., 2021; Goods et al., 2023). Baseline core temperature was defined as the mean core temperature during the early phase of the race (1–5 km), which was used to represent the early-race physiological state before substantial cumulative thermal strain had developed. This phase was selected for temperature baseline estimation rather than for pacing normalization; although pacing may fluctuate during the opening kilometers, averaging core temperature over the 1- to 5-km window was intended to reduce the influence of short-term variability and provide a stable early-race thermal reference. Peak core temperature was defined as the highest value recorded across the entire race. The magnitude of peak core temperature elevation (Δ*T*) was calculated as the difference between peak and baseline core temperature. Using each athlete’s individual decoupling onset (Onset) as an anchor point, mean core temperature was calculated for the pre-decoupling phase (3 km preceding Onset) and the decoupling phase (Onset ± 1 km). The difference between these two values was used to quantify the change in core temperature during the decoupling phase relative to the pre-decoupling phase (Δ*T*core_at–pre_). In addition, the late-race core temperature plateau index was calculated as the proportion of core temperature elevation during the latter stage of the race (15–20 km) relative to the total peak core temperature elevation observed across the entire race.


$$\text{Plateau Index}=\frac{{\text{MeanTemp }}_{15-20\text{km}}-\text{BaselineTemp}}{\text{PeakTemp}-\text{BaselineTemp}}\times 100\%$$

##### 2.5.2.2 Glucose concentration

Glucose concentration was continuously monitored throughout the simulated half-marathon run using a continuous glucose monitoring (CGM) system (GS1 series), with data automatically recorded at 5-min intervals. The clinical performance of this CGM system has been validated in multiple independent evaluations, demonstrating a mean absolute relative difference (MARD) within accepted standards for clinical CGM applications (Yan et al., 2023; Zhao et al., 2025). Based on the time–distance correspondence, glucose data were aligned to the corresponding race distance segments. Mean glucose concentration was calculated for the pre-decoupling phase (3 km preceding Onset) and the decoupling phase (Onset ± 1 km), and the difference between these two values was used to quantify the change in glucose concentration during the decoupling phase relative to the pre-decoupling phase (ΔGlu_at–pre_). In addition, the late-race glucose change (ΔGlu_15–20km_) was calculated as the difference between the mean glucose concentration at the beginning and end of the latter stage of the race (15–20 km).

##### 2.5.2.3 Fluid and electrolyte measures

Fluid and electrolyte variables were assessed using a single-use sweat patch (AbsolutSweat P1). The patch was affixed to the chest region and used to collect localized sweat samples over predefined time- or distance-based intervals during the simulated half-marathon run. Following sample collection, sweat volume was quantified and sweat sodium concentration was analyzed, allowing for the calculation of localized fluid loss and sodium loss within each corresponding interval. To facilitate between-participant comparisons, sweat-related variables were standardized by race distance, and fluid loss per unit distance (mL·km^-1^) and sodium loss per unit distance (mg·km^-1^) were calculated. Using the individual decoupling onset (Onset) as an analytical anchor, changes in distance-normalized fluid loss and sodium loss were computed between the pre-decoupling phase (3 km preceding Onset) and the decoupling phase (Onset ± 1 km), expressed as ΔWater_at–pre_ and ΔNa_at–pre_, respectively. In addition, mean values of these variables during the latter stage of the race (15–20 km) were extracted.

It should be noted that localized sweat sampling methods are primarily intended to reflect relative changes in sweat-related variables and are inherently influenced by factors such as individual sweat rate, sampling site, and environmental conditions. Accordingly, sweat patch data in the present study were used for within-participant, stage-based process comparisons, rather than for estimating whole-body sweat rate or absolute electrolyte loss. This approach is consistent with current methodological recommendations regarding sweat measurement and the application of wearable sweat-sensing technologies (Baker, 2017; Bandiera et al., 2025).

### 2.6 Statistical analysis

All data were examined for distributional characteristics prior to analysis. Given the small sample size and the non-normal distribution of several variables, data were primarily summarized using medians and interquartile ranges to describe process-oriented changes and inter-individual variability in physiological responses during the simulated half-marathon. Physiological data were screened for completeness and plausibility before analysis, and segments affected by transient missing values or clearly implausible recordings were excluded from the relevant analyses. To examine the relationships between heart rate–running speed decoupling characteristics and physiological variables, including core body temperature, glucose concentration, and fluid–electrolyte measures, correlation analyses were performed on key process-based indices. Considering the sample size and distributional characteristics of the data, Spearman’s rank correlation coefficients were used as the primary measure of association. For key Spearman correlation coefficients, 95% confidence intervals were estimated using bias-corrected and accelerated (BCa) bootstrap resampling (10,000 iterations) and are reported in the text. Correlation analyses focused on associations between decoupling timing indices (Onset), decoupling magnitude indices [DM_max_ and late-race decoupling change (15–20 km)], and physiological variables related to thermoregulatory, metabolic, and fluid–electrolyte regulation. For visualization, fitted linear regression lines with 95% confidence intervals of the mean response were displayed in the scatter plots. Given the exploratory design of the study, the small sample size, and the multiple correlation analyses performed across several physiological variables, no formal adjustment for multiple comparisons was applied. Accordingly, *p*-values should be interpreted descriptively, and statistically significant associations should be considered exploratory and hypothesis-generating rather than confirmatory. No *a priori* sample size calculation or formal sensitivity analysis was conducted, and no between-group comparisons or multivariable regression modeling were performed. All statistical tests were two-tailed, with a nominal significance level of *p* < 0.05. Statistical analyses were performed using Python.

## 3 Results

### 3.1 Process-oriented characteristics of heart rate–running speed decoupling during the simulated half-marathon run

**Figure 1:**
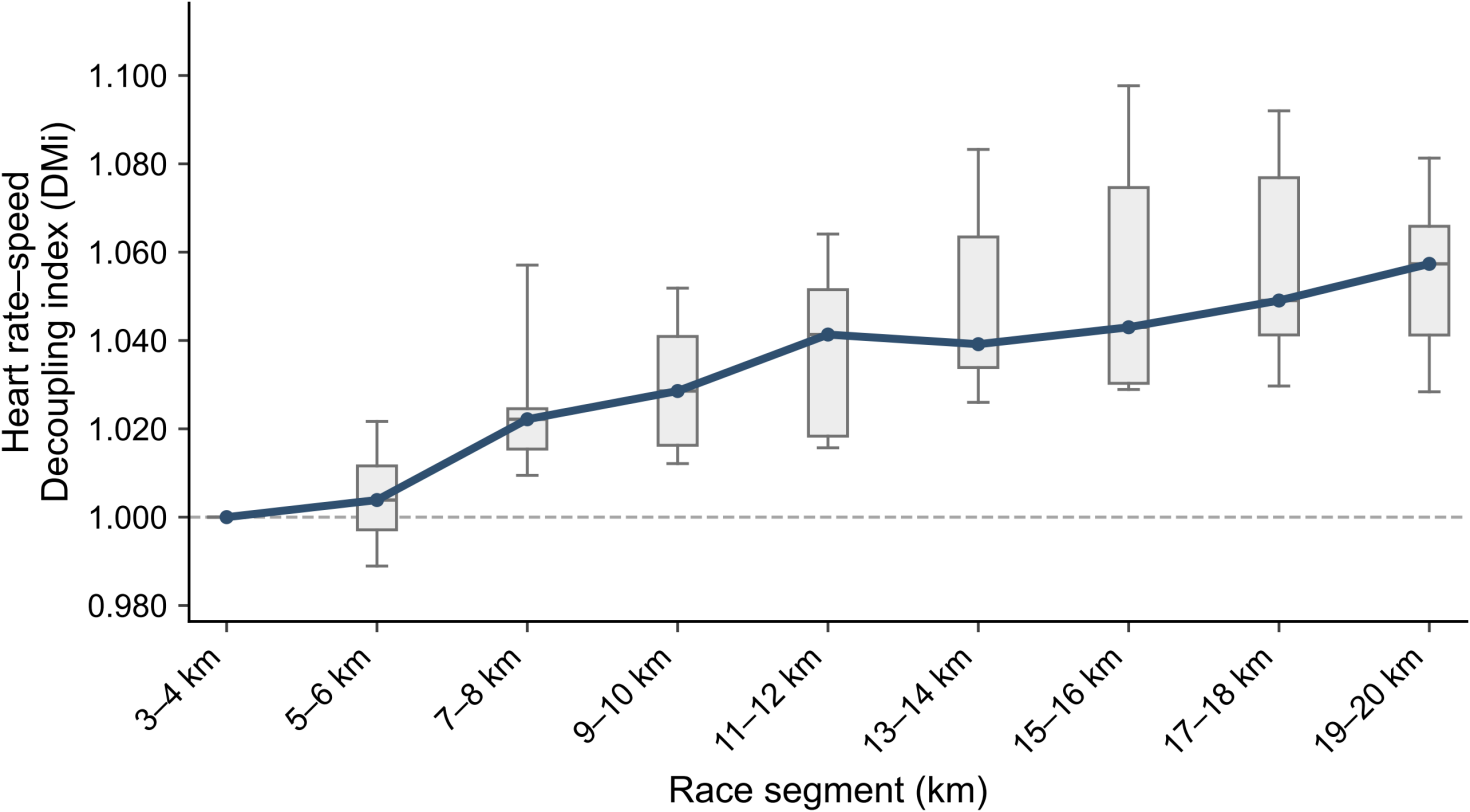
Boxplots represent the distribution of the relative heart rate–running speed load ratio within each distance segment, including the median, interquartile range, and whiskers indicating the 5th–95th percentiles. Solid lines connect the segment-specific medians. The relative decoupling index was normalized to the 3–4 km segment (reference value = 1), and the dashed line indicates the reference baseline.

During the simulated half-marathon run, heart rate–running speed decoupling increased progressively with race distance and showed greater inter-individual variability as the race progressed (**Figure 1**). The median relative decoupling index remained close to 1.00 in the early stage (5–6 km) and rose to approximately 1.06 in the late stage (19–20 km), indicating a progressively greater heart rate response at a given relative external load relative to the early-race reference condition. Segment-specific descriptive statistics are presented in **Table 2A**; across the 2-km race segments, the median relative decoupling index ranged from 1.00 to1.06, and the coefficient of variation ranged from 1.4% to 2.6%, with the greatest between-participant dispersion observed at 15–16 km. Key decoupling characteristics are summarized in **Table 2B**. Decoupling onset showed marked inter-individual variability, with a median onset distance of 10.0 km (interquartile range: 7.0–12.0 km). Maximal decoupling magnitude (DM_max_) also varied substantially among participants, with a median of 7.05% (interquartile range: 5.44%–9.95%). Dispersion in decoupling was particularly pronounced during the latter stage of the race, for which DM_mean-late_ showed a coefficient of variation exceeding 40%, suggesting greater inter-individual differences in decoupling expression during the second half of the simulated half-marathon.

**Table 2A:** Descriptive statistics of the heart rate–running speed relative decoupling index across race segments (2-km segments).

Race segment (km)	*n*	Median (IQR)	CV (%)
5–6	13	1.004 (0.997–1.012)	1.4
7–8	13	1.022 (1.015–1.025)	2.1
9–10	13	1.029 (1.016–1.041)	1.5
11–12	13	1.041 (1.018–1.052)	2.2
13–14	13	1.039 (1.034–1.063)	2.2
15–16	13	1.043 (1.030–1.075)	2.6
17–18	13	1.049 (1.041–1.077)	2.3
19–20	13	1.057 (1.041–1.066)	1.9

Data are presented as median (interquartile range). CV, coefficient of variation.

**Table 2B:** Descriptive statistics of key internal–external load decoupling characteristics.

Variable	*n*	Median (IQR)	CV (%)
Decoupling onset (km)	13	10.0 (7.0–12.0)	28.6
Maximal decoupling magnitude (DM_max_, %)	13	7.05 (5.44–9.95)	35.9
DM_mean-late_ (%; 15–20 km)	13	4.18 (4.03–6.79)	40.7

Decoupling onset was identified based on a predefined threshold applied to the relative decoupling index (DMi). DMmax represents the maximal relative decoupling deviation from the reference segment, whereas DMmean-late represents the mean decoupling level during the latter stage of the race (15–20 km). Data are presented as median (interquartile range). CV, coefficient of variation.

### 3.2 Decoupling onset and magnitude characteristics and their associations with physiological variables

#### 3.2.1 Associations between decoupling onset and physiological variables

**Figure 2:**
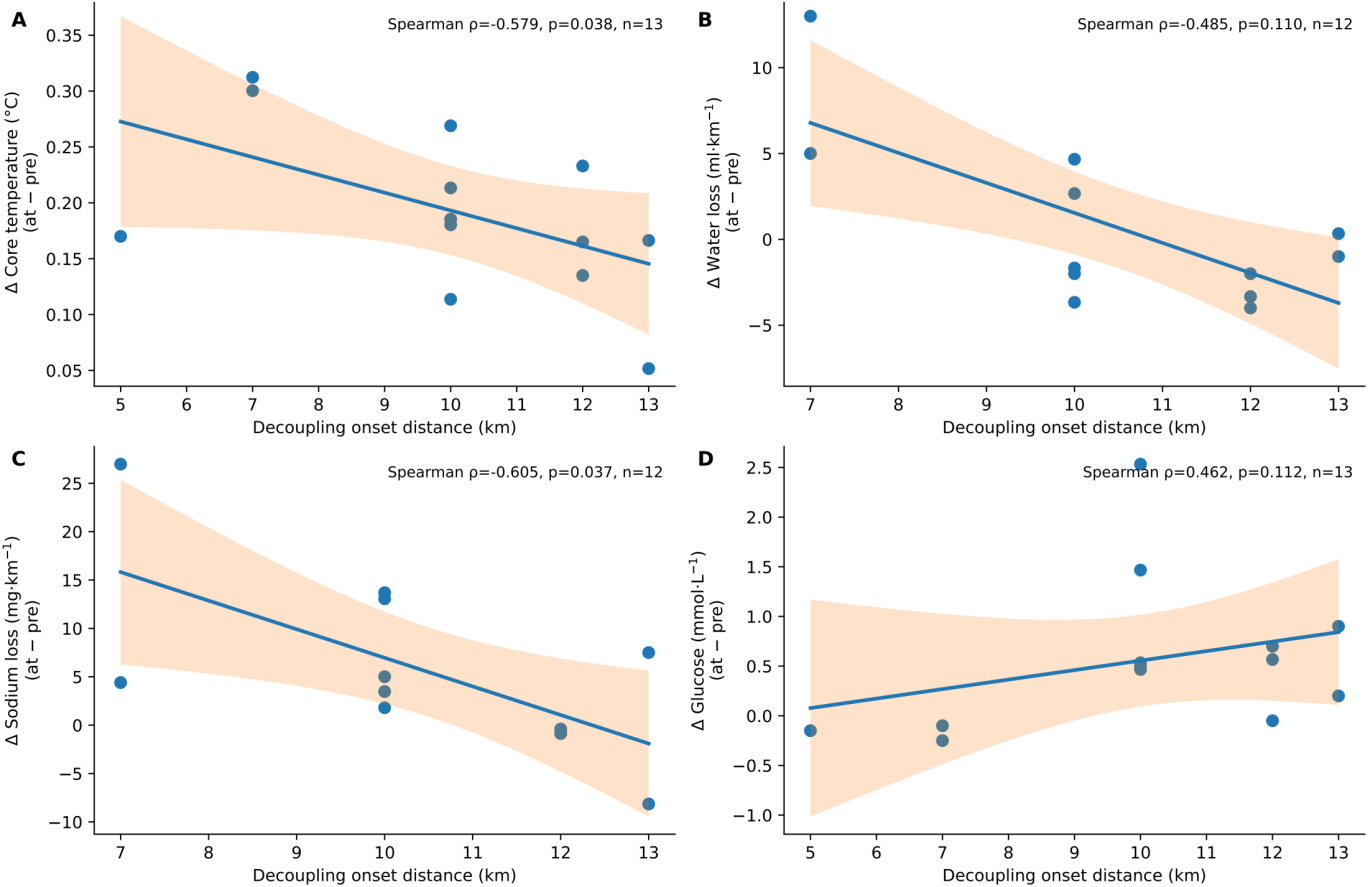
Scatter plots showing the associations between individual heart rate–running speed decoupling onset distance (Onset, km) and changes in multiple physiological variables across the decoupling window: **(A)** change in core body temperature, **(B)** change in fluid loss per unit distance, **(C)** change in sodium loss per unit distance, and **(D)** change in glucose concentration. Solid lines indicate fitted linear regression lines for visualization only, with shaded areas representing 95% confidence intervals. Associations were quantified using Spearman's rank correlation coefficients. Each point represents one participant. Sample sizes differed slightly across panels because of missing data (A and D: n = 13; B and C: n = 12).

Using each athlete’s individual decoupling onset (Onset, km) as an anchor point, changesin core body temperature, glucose concentration, and fluid- and electrolyte-related indices were examined between the pre-decoupling phase (3 km preceding Onset) and the decoupling phase (Onset ± 1 km). For each variable, changes were expressed as the difference between the decoupling phase and the pre-decoupling phase (Δ_at–pre_; **Table 3**). Correlation analyses showed that decoupling onset was significantly negatively associated with the change in core body temperature [Spearman ρ = −0.579, 95% CI (−0.897, −0.024), *p* = 0.038, *n* = 13; **Figure 2A**] and was negatively associated with the change in sodium loss per unit distance [ρ = −0.605, 95% CI (−0.937, 0.165), *p* = 0.037, *n* = 12; **Figure 2C**]. In contrast, no significant association was observed between decoupling onset and the change in fluid loss per unit distance [ρ = −0.485, 95% CI (−0.890, 0.277), *p* = 0.110, *n* = 12; **Figure 2B**], and the association with the change in glucose concentration did not reach statistical significance [ρ = 0.462, 95% CI (−0.286, 0.832), *p* = 0.112, *n* = 13; **Figure 2D**]. Descriptive statistics for these variables are provided in **Table 3**.

**Table 3:** Onset-related physiological changes during the decoupling phase.

Variable	Median (IQR)
Δ*T*core_at–pre_ (°C)	0.18 (0.17–0.23)
ΔGlu_at–pre_ (mmol·L^-1^)	0.50 (0.20–1.47)
ΔNa_at–pre_ (mg·km^-1^)	3.47 (−0.40–7.50)
ΔWater_at–pre_ (mL·km^-1^)	−1.33 (−2.33–3.17)

#### 3.2.2 Associations between maximal decoupling magnitude and physiological variables

**Figure 3:**
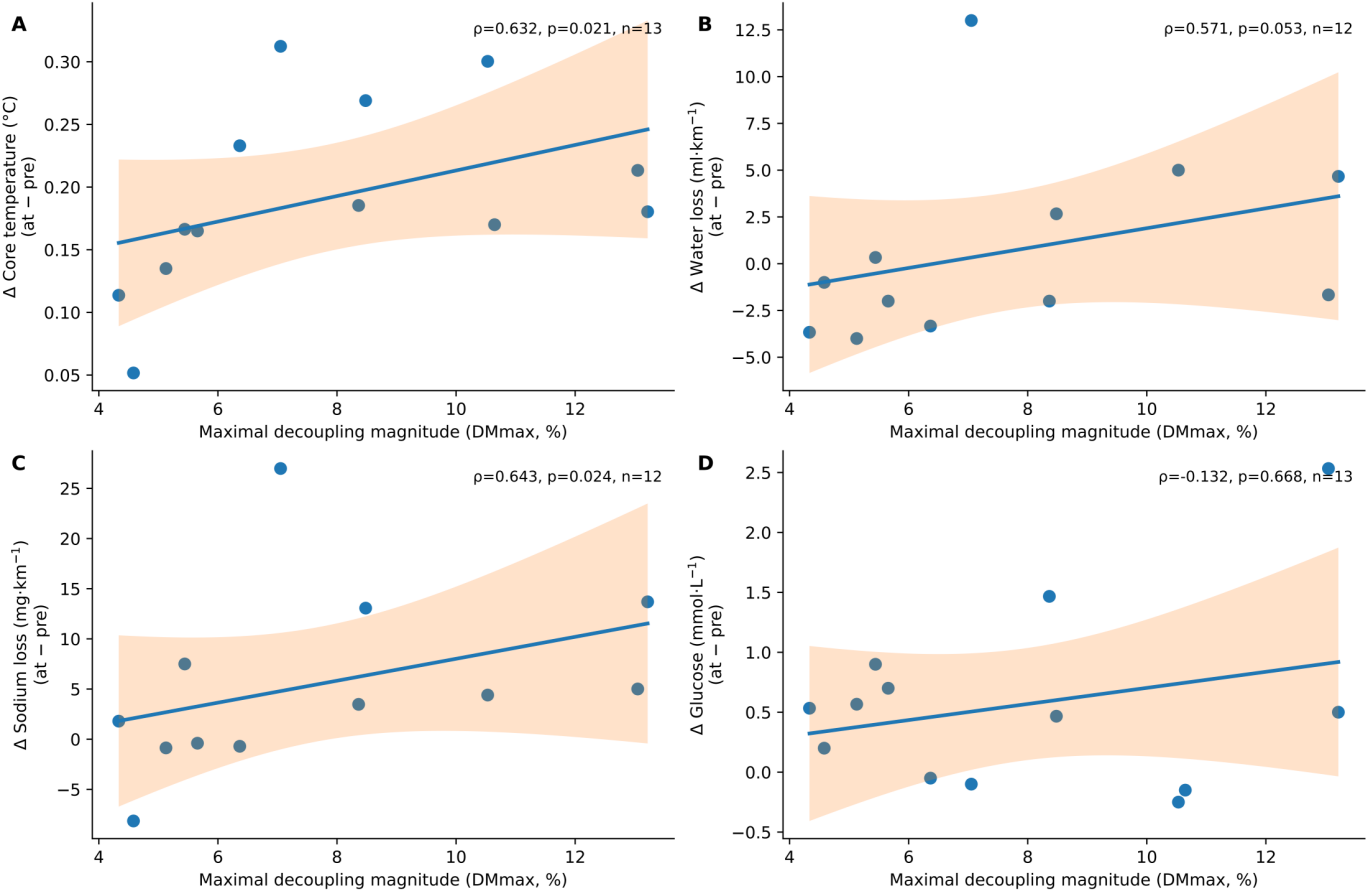
Scatter plots showing the associations between maximal heart rate–running speed decoupling magnitude (DMmax, %) and changes in multiple physiological variables during the decoupling phase relative to the pre-decoupling phase: **(A)** change in core body temperature, **(B)** change in fluid loss per unit distance, **(C)** change in sodium loss per unit distance, and **(D)** change in glucose concentration. Solid lines indicate fitted linear regression lines for visualization only, with shaded areas representing 95% confidence intervals. Associations were quantified using Spearman's rank correlation coefficients. Each point represents one participant. Sample sizes differed slightly across panels because of missing data (**A, D**: n = 13; **B, C**: n = 12).

Additional analyses examined the associations between maximal decoupling magnitude(DM_max_) and concurrent physiological changes during the decoupling phase relative to the pre-decoupling phase (Δ_at–pre_; **Table 3**; **Figure 3**). Correlation analyses showed that DM_max_ was positively associated with the change in core body temperature [Spearman ρ = 0.632, 95% CI (−0.014, 0.908), *p* = 0.021, *n* = 13; **Figure 3A**] and with the change in sodium loss per unit distance [ρ = 0.643, 95% CI (0.199, 0.949), *p* = 0.024, *n* = 12; **Figure 3C**]. In contrast, the association between DM_max_ and the change in fluid loss per unit distance did not reach statistical significance [ρ = 0.571, 95% CI (0.040, 0.879), *p* = 0.053, *n* = 12; **Figure 3B**], and no significant association was observed between DM_max_ and the change in glucose concentration [ρ = −0.132, 95% CI (−0.631, 0.548), *p* = 0.668, *n* = 13; **Figure 3D**]. Descriptive statistics for the corresponding physiological changes are presented in **Table 3**.

#### 3.2.3 Associations between late-race decoupling changes and physiological variables

Late-race decoupling was further characterized using the change in the relative decoupling indexduring the 15- to 20-km segment (ΔDecoupling_15–20 km_). Descriptive statistics for late-race decoupling and the corresponding physiological variables are presented in **Table 4**, and correlation results are presented in **Table 5**. Correlation analyses showed that ΔDecoupling_15–20 km_ was not significantly associated with the late-race core temperature plateau index (Spearman ρ = −0.088, *p* = 0.775, *n* = 13), fluid loss per unit distance (ρ = −0.039, *p* = 0.900, *n* = 13), or sodium loss per unit distance (ρ = −0.037, *p* = 0.929, *n* = 13). In contrast, ΔDecoupling_15–20 km_ was significantly positively associated with the change in glucose concentration during the late-race phase (ρ = 0.599, *p* = 0.031, *n* = 13).

**Table 4:** Descriptive statistics of late-race decoupling changes and associated physiological variables.

Variable	Median (IQR)	*n*
ΔDecoupling_15–20 km_	0.002 (−0.009–0.008)	13
Late-race core temperature plateau index (15–20 km)	0.467 (0.412–0.800)	13
Late-race fluid loss per unit distance (mL·km^-1^)	38.000 (34.333–43.667)	13
Late-race sodium loss per unit distance (mg·km^-1^)	51.788 (41.553–65.887)	13
ΔGlucose (mmol·L^-1^)	−0.350 (−0.850–0.250)	13

Data are presented as median (interquartile range, IQR). ΔDecoupling15–20 km represents the change in the relative decoupling index across the 15–20 km segment. The late-race core temperature plateau index reflects the degree of core temperature stabilization relative to the total elevation observed across the entire race. Fluid and sodium loss variables were normalized per unit distance (km). ΔGlucose represents the difference in mean glucose concentration between the beginning and end of the 15–20 km segment.

**Table 5:** Correlation analysis between late-race decoupling changes and physiological variables.

Decoupling variable	Physiological variable	*n*	Spearman’s ρ	*P*-value
ΔDecoupling_15–20 km_	Late-race core temperature plateau index (15–20 km)	13	−0.088	0.775
ΔDecoupling_15–20 km_	Late-race fluid loss per unit distance (mL·km^-1^)	13	−0.039	0.900
ΔDecoupling_15–20 km_	Late-race sodium loss per unit distance (mg·km^-1^)	13	−0.037	0.929
ΔDecoupling_15–20 km_	ΔGlucose (mmol·L^-1^)	13	0.599	0.031

Spearman's rank correlation coefficients (ρ) were used to assess associations between late-race decoupling changes and physiological variables.ΔDecoupling15–20 km represents the change in the relative decoupling index across the 15–20 km segment. ΔGlucose denotes the change in mean glucose concentration between the beginning and end of the 15–20 km segment.

## 4 Discussion

### 4.1 Interpreting decoupling as a process-oriented indicator

Under a simulated half-marathon race condition, the present study used the heart rate–running speed internal–external load ratio to continuously characterize decoupling in adolescent endurance athletes during prolonged exercise. The results showed a progressive increase in the relative decoupling index across the race, together with marked inter-individual variability. These findings suggest that, as endurance load accumulates, the relationship between heart rate and running speed is not stable but changes dynamically over the course of the race (Coyle and González-Alonso, 2001; Billat et al., 2020). In previous research and applied practice, heart rate–power or heart rate–pace decoupling has commonly been used as an indicator of aerobic endurance capacity or pacing stability, typically quantified by comparing changes in the Pa: Hr (or Pw: Hr) ratio between the first and second halves of exercise. Smaller degrees of decoupling have generally been interpreted as an empirical sign of more stable internal–external load coupling during prolonged exercise (Billat et al., 2020; Maunder et al., 2021). From an applied perspective, however, smaller decoupling does not necessarily imply a more favorable performance outcome or superior mechanical efficiency. Rather, it may reflect a greater ability to maintain external output with less progressive internal physiological drift, which is more consistent with better durability or pacing stability. However, this binary phase-comparison approach tends to conceptualize decoupling as a result-based or threshold phenomenon. As a result, it may overlook both the progressive nature of decoupling and the individualized trajectories that can emerge under real-world race conditions or prolonged endurance loading (Smyth et al., 2022).

By applying a distance-segmented and within-individual normalization approach, the present study suggests that decoupling is better understood as a process-oriented response rather than as a transient or abrupt shift. In this sample, decoupling developed progressively with cumulative load and showed substantial inter-individual heterogeneity in both onset and magnitude. This perspective aligns with contemporary durability theory, which emphasizes an athlete’s ability to resist fatigue-induced deterioration in physiological and performance variables during prolonged exercise. In other words, durability reflects the capacity to maintain performance under fatigue, rather than relying only on assessments made in a fresh state (Maunder et al., 2021; Matomäki et al., 2023). From this perspective, decoupling onset may be interpreted as a temporal expression of durability, whereas decoupling magnitude may reflect the severity of durability loss. This framework may help explain how different decoupling characteristics are related to multiple physiological regulatory responses during prolonged endurance exercise.

### 4.2 Associations between decoupling timing and thermoregulatory and electrolyte regulatory responses

The present findings indicate that the timing of heart rate–running speed decoupling onset varied substantially among individuals and was statistically associated with thermoregulatory and electrolyte-related physiological burdens during the initial stage of decoupling. Specifically, decoupling onset was negatively correlated with both the magnitude of core body temperature elevation and the change in sodium loss per unit distance during the decoupling phase. This pattern suggests that athletes with earlier decoupling onset tended to show greater increases in core temperature and sodium loss at the stage when decoupling first emerged.

During prolonged endurance exercise, the progressive elevation of core body temperature is widely recognized as a key physiological stressor that can affect cardiovascular regulatory stability. As exercise duration increases, greater thermoregulatory demands increase skin blood flow demands and are often accompanied by reductions in venous return and stroke volume. Heart rate then increases in a compensatory manner to help maintain cardiac output, giving rise to the classical phenomenon of cardiovascular drift (Coyle and González-Alonso, 2001; Wingo et al., 2012). Under such conditions, even when external load (e.g., running speed) remains relatively stable, heart rate may continue to rise over time, resulting in a progressive shift in the heart rate–running speed relationship. From this perspective, heart rate–running speed decoupling may be viewed as one observable expression of thermoregulatory-related cardiovascular strain during prolonged exercise. Compared with previous studies that mainly emphasized decoupling magnitude or pre–post race differences, the present study expanded the analytical focus by introducing decoupling onset as a process-oriented indicator, thereby addressing when decoupling begins to emerge. The findings suggest that decoupling onset is not simply random variation, but is associated with the thermal and electrolyte-related physiological load present during the initial phase of decoupling. Accordingly, the timing of decoupling onset may reflect inter-individual differences in the ability to regulate heat stress and fluid–electrolyte perturbations during the early to middle stages of prolonged exercise, rather than merely representing an outcome of late-race fatigue. It should be emphasized that the associations observed in the present study should not be interpreted as evidence of direct causality between thermoregulatory or electrolyte changes and decoupling. In endurance exercise, thermoregulatory, cardiovascular, and performance responses are tightly coupled, and the observed relationships are more likely to reflect the synchronous temporal evolution of multiple physiological regulatory processes, including core temperature, glucose availability, and fluid–electrolyte balance. Nevertheless, from a process-oriented perspective, these findings provide insight into when decoupling begins to emerge and support the interpretation of decoupling onset as a potential marker of inter-individual differences in endurance durability.

### 4.3 Stage-specific contributions of different physiological systems to the development of decoupling

The present findings indicate that the associations between heart rate–running speed decoupling and multiple physiological variables—including core body temperature, glucose concentration, and fluid–electrolyte regulation—were not uniform across the race, but differed by exercise stage. Specifically, with respect to decoupling timing and maximal decoupling magnitude, the observed associations were primarily with indices related to thermoregulatory and electrolyte-related load. In contrast, during the latter stage of the race, changes in decoupling magnitude were not significantly associated with core temperature, fluid loss, or sodium loss, but were positively associated with changes in glucose concentration. As an important physiological marker related to energy supply regulation during endurance exercise, glucose dynamics are influenced by a complex interplay of factors, including hepatic glycogen output, skeletal muscle glucose uptake, hormonal regulation, exercise intensity, and psychological stress, and therefore typically exhibit pronounced nonlinearity and inter-individual variability (Flockhart and Larsen, 2024). Compared with thermoregulatory-related indices, glucose concentration is also more susceptible to acute modulation by feeding behavior and nutritional strategies (Burke and Jeukendrup, 2014; Jeukendrup, 2017). Accordingly, during the early to middle stages of endurance exercise, glucose changes may not show a stable or linear correspondence with cumulative load or with alterations in heart rate–running speed coupling. This characteristic may help explain why, in the present study, glucose changes were not significantly associated with decoupling timing or maximal decoupling magnitude. By contrast, during the late stage of the race, when thermoregulatory and fluid–electrolyte responses may approach relatively stable or plateau-like states, inter-individual differences may become more clearly expressed in glucose-related physiological responses. The observed significant association between late-race decoupling changes and glucose dynamics suggests that further changes in internal–external load coupling during this stage may co-occur with fluctuations in energy availability. This finding should not be interpreted as evidence that glucose changes directly drive decoupling evolution. Rather, it more likely reflects the temporal co-evolution of internal physiological cost and glucose-related physiological signals during the later stages of prolonged endurance exercise.

However, fluid- and electrolyte-related variables, particularly sodium loss, did not exhibit significant associations with changes in decoupling magnitude during the latter stage of the race. This pattern may be related to the pronounced individual variability and context dependency of these measures. Previous studies have shown that both sweat rate and sweat sodium concentration display substantial within- and between-individual variability and are influenced by multiple factors, including environmental conditions, heat acclimation status, biological maturity, and hydration or fueling strategies (Baker, 2017). Once individual sweating patterns and electrolyte loss rates become relatively stable late in the race, their incremental changes may contribute less to explaining additional variation in decoupling. Taken together, the present findings support a stage-specific regulatory framework. During the early to middle stages of prolonged endurance exercise, decoupling timing and maximal decoupling magnitude appeared to co-vary primarily with thermoregulatory- and electrolyte-related physiological strain. During the latter stage, changes in decoupling magnitude appeared to co-vary with glucose-related physiological signals. This perspective supports a more nuanced interpretation of heart rate–running speed decoupling by highlighting that the physiological information reflected by this index may differ across exercise stages.

From an applied perspective, these findings suggest that earlier decoupling onset or greater maximal decoupling may justify closer monitoring of thermal strain and electrolyte-related load during the early to middle stages of prolonged exercise, whereas continued decoupling development in the latter stage may warrant greater attention to energy-supply-related factors and late-race fueling support. These implications remain exploratory and should not be interpreted as evidence of intervention efficacy or direct causality. Nevertheless, they may still provide practitioners with evidence-informed guidance for interpreting stage-specific physiological strain during prolonged endurance exercise. In addition, the internal cost of maintaining running speed may also be influenced by biomechanical determinants of running economy, including cadence, vertical displacement, lower-limb stiffness, and musculotendinous properties. Current evidence suggests that these variables explain a modest but practically meaningful proportion of the between-individual variance in running economy, with lower vertical displacement and higher vertical or leg stiffness showing more consistent associations with lower oxygen or energy cost during running, while musculotendinous properties may also contribute to economical running patterns (van Hooren et al., 2024; Liu et al., 2022; Arampatzis et al., 2006). Accordingly, future studies that integrate physiological, biomechanical, and neuromuscular monitoring may help provide a more complete explanation of heart rate–running speed decoupling.

### 4.4 Limitations

The present study used a simulated half-marathon setting to explore the process-oriented characteristics of heart rate–running speed decoupling in adolescent endurance athletes during prolonged exercise, as well as its associations with core body temperature, glucose concentration, and fluid–electrolyte-related variables. Although this design enabled the collection of continuous internal and external load data under relatively controlled conditions and allowed dynamic changes in decoupling to be characterized across the exercise process, several limitations should be considered when interpreting the findings. First, the sample size was small, and the study population consisted exclusively of adolescent endurance athletes within a specific age range. Adolescents may differ from adult or elite endurance athletes in physiological maturity, thermoregulatory capacity, metabolic regulation strategies, and training background. Accordingly, the present findings should be interpreted as exploratory and population-specific, and they should not be directly extrapolated to athletes of other age groups or competitive levels. Future studies with larger samples, broader age ranges, both sexes, and different performance levels are needed to extend these observations. Second, although the simulated half-marathon design allowed standardized testing procedures and the synchronous collection of multiple physiological variables, it could not fully reproduce the complex and dynamic conditions of real-world competition. Environmental temperature, humidity, wind speed, competitive pressure, and real-time tactical adjustments may all influence heart rate responses, pacing strategies, and decoupling characteristics. Therefore, the ecological validity of the present findings should be interpreted with caution. Under greater heat stress or stronger competitive demands, the timing and magnitude of heart rate–running speed decoupling may show different patterns, which were not systematically examined in the present study. Third, heart rate was used as the primary indicator of internal load. Although heart rate–running speed decoupling is widely used to reflect internal–external load coupling during endurance exercise, heart rate is influenced by multiple factors, including hydration status, sympathetic nervous system activity, psychological stress, and environmental conditions. During prolonged endurance exercise, heart rate kinetics do not always align precisely with metabolic responses such as oxygen uptake or ventilatory dynamics. Accordingly, the decoupling index should be interpreted as an integrated physiological marker rather than as evidence of a single underlying mechanism. Future studies incorporating respiratory metabolic variables or muscle metabolic indicators may help further clarify the physiological basis of decoupling. Finally, although no additional fluid intake was permitted during the simulated half-marathon, analyses of glucose dynamics and fluid–electrolyte regulation did not include pre-exercise nutritional or hydration status, or other individual fueling-related factors, as primary covariates. Previous research has shown that carbohydrate intake and sodium-containing fluid ingestion can substantially influence glucose dynamics and fluid balance during endurance exercise. Therefore, even under controlled in-race fluid intake, uncontrolled pre-exercise status and fueling-related factors may still have contributed to inter-individual variability in these physiological responses and may have attenuated the observed associations with decoupling characteristics. Future studies may benefit from more rigorous control or systematic manipulation of pre-exercise nutritional and hydration status to more clearly examine the roles of energy supply and fluid–electrolyte regulation in the development of decoupling.

It should also be emphasized that the correlation-based analytical approach used in the present study was intended to examine process-oriented associations rather than to establish causal relationships. During endurance exercise, thermoregulatory, metabolic, cardiovascular, and fluid–electrolyte processes are tightly coupled and dynamically interact over time. Accordingly, the associations observed here are more likely to reflect the synchronous temporal evolution of multiple physiological regulatory processes than direct causal effects. Future research using longitudinal designs, experimental or intervention-based approaches, or multivariable modeling frameworks may help further validate and extend these findings and clarify the mechanisms underlying heart rate–running speed decoupling.

## 5 Conclusion

This study investigated adolescent endurance athletes under a simulated half-marathon running condition and, from a process-oriented perspective, systematically examined the characteristics of heart rate–running speed decoupling and its associations with multiple physiological regulatory responses, including core body temperature, glucose concentration, and fluid–electrolyte balance. The findings demonstrate that, under prolonged endurance loading, heart rate–running speed decoupling is a common phenomenon that progressively increases as the race unfolds, with both decoupling timing and decoupling magnitude exhibiting marked inter-individual variability. These results indicate that the internal–external load coupling state is not stable, but dynamically evolves with cumulative exercise load. Importantly, different process-related features of decoupling showed stage-specific associations with physiological regulation. The timing of decoupling onset and the maximal decoupling magnitude were primarily associated with thermoregulatory and electrolyte regulatory burden during the initial phase of decoupling, suggesting that heat stress and fluid–electrolyte regulation may play an important role in shaping internal–external load relationships during the early to middle stages of endurance exercise. In contrast, during the latter stage of the race, changes in decoupling magnitude were not significantly associated with core temperature or fluid–electrolyte indices, but were significantly associated with changes in glucose concentration, suggesting that late-race decoupling may co-vary with physiological signals related to energy supply regulation.

Overall, the present study supports the interpretation of heart rate–running speed decoupling as a durability-related indicator with clear process-oriented properties, whose distinct characteristic dimensions reflect stage-specific contributions of multiple physiological regulatory systems, including core body temperature, glucose availability, and fluid–electrolyte balance, during prolonged endurance exercise. These findings provide novel empirical evidence for understanding the regulatory strategies and durability characteristics of adolescent endurance athletes from a dynamic, multi-system perspective under sustained loading conditions, and offer a valuable reference for future research and applied monitoring approaches that integrate exercise progression into the analysis of internal–external load relationships.

## Data Availability

The raw data supporting the conclusions of this article will be made available by the authors, without undue reservation.
