# Supplementary material for: Physiological associations with heart rate–speed decoupling during a half-marathon in adolescent endurance runners
Source: Front Physiol. 2026 Apr 29;17:1807399. doi: 10.3389/fphys.2026.1807399 (PMC13167590; doi:10.3389/fphys.2026.1807399)
Supplement: Supplementary file 1 [file Table1.docx]

Supplementary Material

# Supplementary Tables

Table S1. Environmental conditions on test days (descriptive statistics)

| Category | Variable | Value (mean ± SD) |
| --- | --- | --- |
| Environmental conditions | Air temperature (°C) | 19.7 ± 0.6 |
|  | Relative humidity (%) | 61.3 ± 1.8 |
|  | Wind speed (m·s⁻¹) | 1.49 ± 0.85 |

Environmental conditions were recorded every 15 minutes on each test day as background information and were not included in the primary statistical modeling or hypothesis testing.

Table S2. Summary of within-segment speed variation in early-race segments(km·h⁻¹)

| **Segment** | **Median** | **IQR** |
| --- | --- | --- |
| 1–2 km | 0.33 | 0.22–0.45 |
| 3–4 km | 0.12 | 0.07–0.19 |
| 5–6 km | 0.25 | 0.12–0.37 |

Within-segment speed variation was calculated as the absolute difference in running speed between the two consecutive 1-km splits within each 2-km segment (i.e., |km1 − km2|, |km3 − km4|, and |km5 − km6|). Smaller values indicate greater pacing stability within the segment. Within-segment speed variation was lower in the 3–4 km segment than in the 1–2 km segment in 12 of 13 athletes.
